# Persistent Vomiting and Weight Loss Leading to the Diagnosis of Barrett’s Esophagus in an Adolescent

**DOI:** 10.7759/cureus.7151

**Published:** 2020-03-01

**Authors:** Alyssa Lorenze, Collin John, Brian D Riedel, Linda S Nield

**Affiliations:** 1 Pediatrics, West Virginia University School of Medicine, Ruby Memorial Hospital, Morgantown, USA; 2 Internal Medicine, West Virginia University, Morgantown, USA; 3 Pediatrics, West Virginia University, Morgantown, USA

**Keywords:** esophageal stricture, barrett’s esophagus, dysphagia, reflux, vomiting, developmental delay, peptic ulcer

## Abstract

Barrett’s esophagus in children with peptic strictures has not been well characterized, and its prevalence is unknown. We report a case of peptic esophageal stricture with Barrett’s esophagus in an adolescent patient who presented with dysphagia with recurrent episodes of vomiting and limited medical history.

A 13-year-old male with mild intellectual disability was transferred to our facility due to a two-month history of dysphagia with recurrent episodes of vomiting and intolerance to both solids and liquids. Physical examination and laboratory values were within normal limits, including complete blood count and differential, serum electrolytes, glucose, amylase, lipase, liver and kidney function tests, and thyroid-stimulating hormone level. Barium esophagram revealed persistent focal narrowing of the proximal and mid-esophagus. An esophageal endoscopy revealed a snug circumferential stricture and biopsy consistent with erosive esophagitis. The patient was started on high dose pantoprazole and underwent serial endoscopic guided balloon dilations with marked improvement in symptoms.

Peptic stricture with Barrett’s esophagus is rare in children. It should be included in the differential diagnosis of a child with the common symptom of vomiting in the setting of developmental delay. Vigorous treatment with endoscopic balloon dilation and proton pump inhibitors is necessary to prevent the progression into adenocarcinoma.

## Introduction

Barrett’s esophagus is a premalignant condition of unknown etiology rarely reported in the pediatric population. Chronic gastrointestinal reflux seems to play the biggest role in its pathogenesis with other risk factors, including intellectual disability, developmental delay, cerebral palsy, gastric tube placement, and esophageal atresia [1-4]. Patients with Barrett’s esophagus have a higher incidence of complications, including strictures, ulceration, and the potential development of adenocarcinoma. Pediatric patients, particularly those with intellectual disabilities, often have silent or non-specific symptoms, predisposing them to late presentation. Early recognition of high-risk children is crucial in the initial investigation and work up. 

Barrett’s esophagus in children with peptic strictures has not been well characterized, and its prevalence is not known [1]. We report a case of peptic esophageal stricture with Barrett’s esophagus in an adolescent patient with mild intellectual disability who presented with dysphagia and recurrent episodes of vomiting. Prompt, appropriate treatment can significantly improve the quality of life and lead to proper surveillance.

## Case presentation

A 13-year-old Caucasian male with mild intellectual disability, attention deficit hyperactivity disorder and constipation, was transferred to our tertiary care facility due to a two-month history of dysphagia with recurrent episodes of vomiting. Initially, the patient vomited only after ingesting solids, but this progressed to include liquids. Further history was difficult to obtain due to the child’s intellectual disability and speech delay. History from various family members as well as caregivers at the psychiatric institution from where he was transferred asserted that the boy also experienced self-induced vomiting on occasion, heartburn, and a seven-pound weight loss in one week. His medications included polyethylene glycol and ranitidine. 

**Figure 1:**
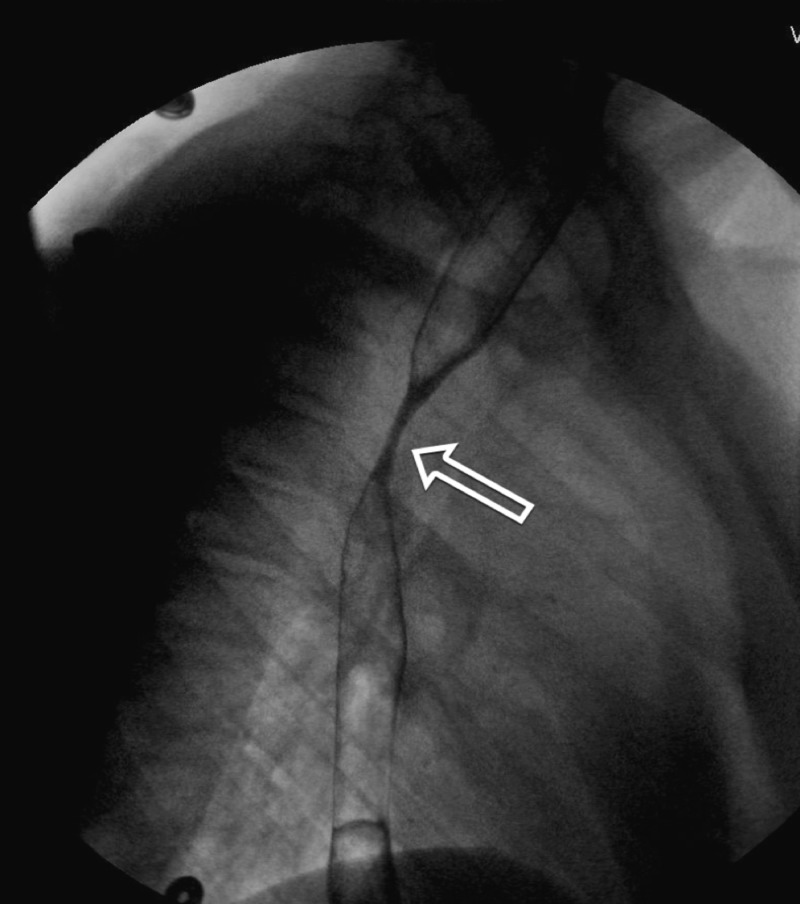
Barium esophagram showing stricture of the esophagus (arrow)

His physical examination upon admission was completely unremarkable. Laboratory values were within normal limits, including complete blood count and differential, serum electrolytes, glucose, amylase, lipase, liver and kidney function tests, and thyroid-stimulating hormone level. Computed tomography (CT) scan of the abdomen, which initially was done to rule out any obstructive causes for his symptoms, revealed stool retention. Despite treatment with polyethylene glycol, which completely resolved the stool retention, he continued to have immediate postprandial emesis. Additional diagnostic testing with barium esophagram was performed, which revealed: “focal persistent narrowing of the proximal and mid-esophagus” (Figure 1). 

**Figure 2:**
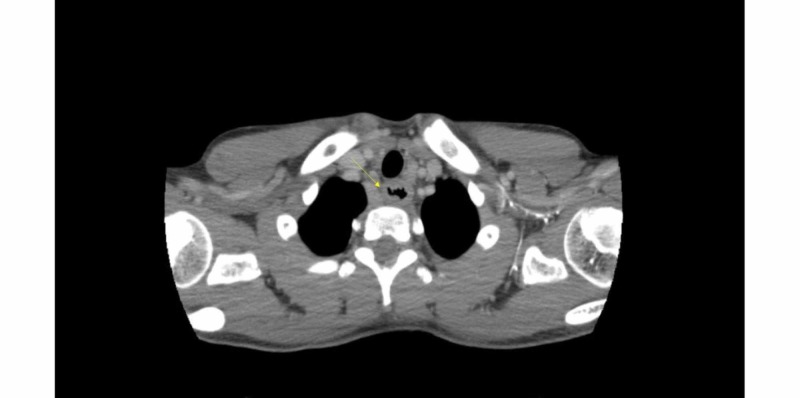
Computed tomography angiography showing circumferential thinking of the esophagus (arrow)

**Figure 3:**
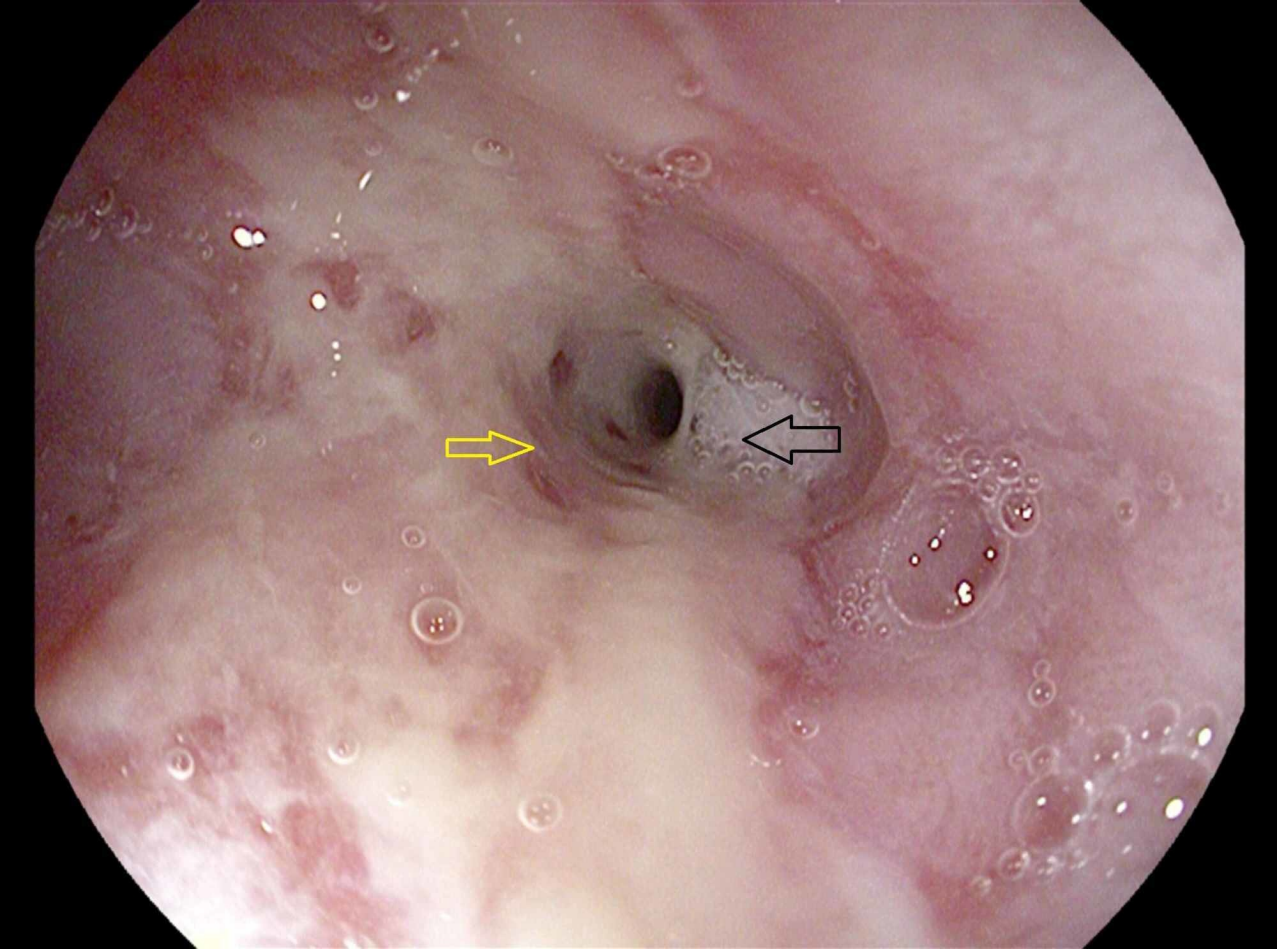
Endoscopy of the esophagus showing erosive esophagitis (yellow arrow) with stricture (black arrow)

External compression of the esophagus was ruled-out with a CT angiogram of the chest, which showed circumferential thickening of the esophagus (Figure 2). Pediatric gastroenterology was consulted, and an esophageal endoscopy with biopsy was performed, which showed a snug circumferential stricture with a diameter of approximately 6 mm at 24 cm from the incisors (Figure 3). 

**Figure 4:**
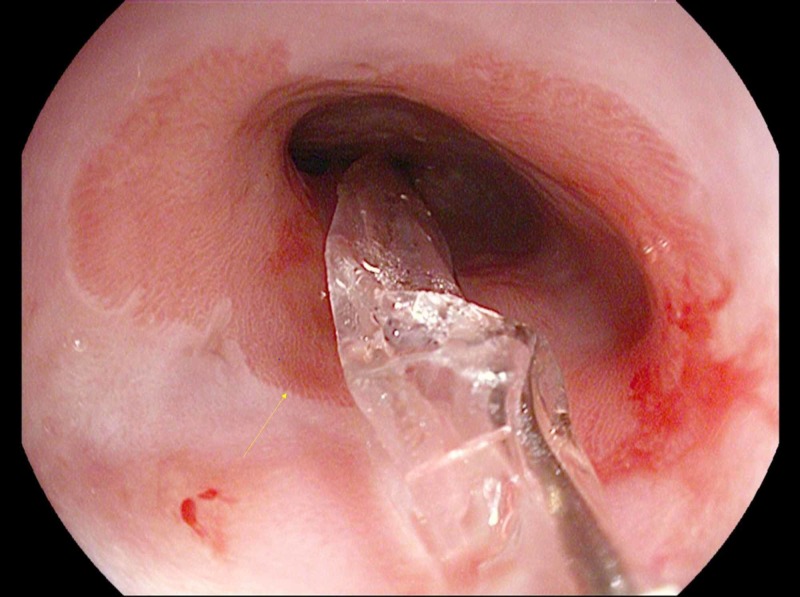
Balloon dilator in place and Barrett’s mucosa (arrow showing Barrett’s mucosa)

The biopsy results indicated the presence of erosive esophagitis. During the same sitting, endoscopic guided balloon dilation was done to 8 mm. The procedure was well tolerated without any complications, and he was discharged on high dose pantoprazole (40 mg twice daily) for erosive esophagitis with peptic esophageal stricture with Barret’s esophagus (Figure 4). 

**Figure 5:**
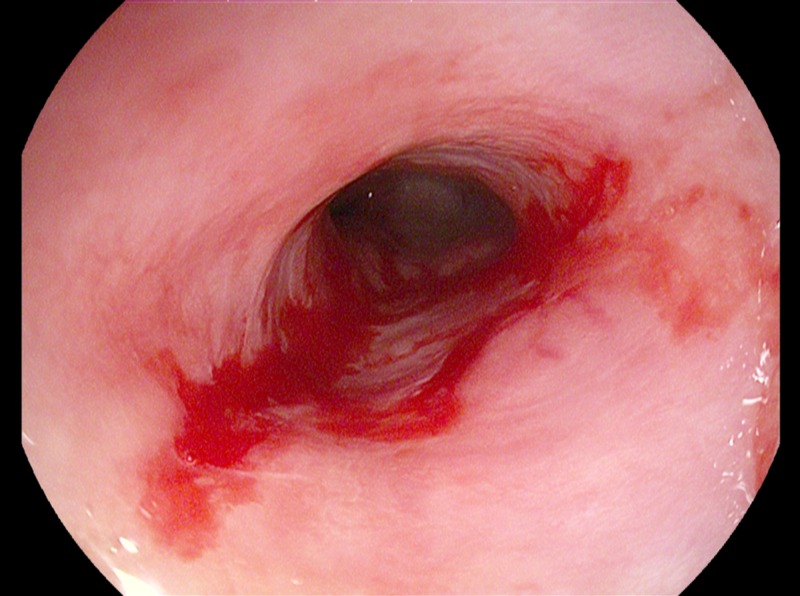
Esophagus after final dilation to 15 mm

Serial repeat dilations were done at three- to four-week intervals with resultant dilation of the esophagus to 15 mm (Figure 5). Since the procedures, the boy tolerated an advancing diet. Vomiting episodes resolved, and consistent weight gain occurred.

## Discussion

Our patient presented with dysphagia with recurrent episodes of vomiting and limited medical history. Initially, the differential diagnosis included more common entities such as gastroesophageal reflux disease (GERD), cyclic vomiting syndrome, or rumination syndrome. Barium esophagram aided the diagnosis of esophageal stricture. Our patient also had Barrett’s esophagus, which appeared on endoscopy and confirmed by pathology. Diagnosis of peptic stricture can usually be suspected with a careful history but should be confirmed with a barium esophagram followed by endoscopy with biopsies [2].

Though esophageal stricture presents in children, it is relatively rare. In the primary care setting, esophageal strictures are most commonly peptic strictures, which develop in 8% of individuals with GERD [3,4]. Although they can occur in any age group, peptic strictures tend to occur most often in elderly patients [5]. The occurrence of benign esophageal stricture increases with age, at a mean of 59 years [6]. Potential complications include a chronic relapsing course, an increased risk of food impaction, an increased risk for pulmonary aspiration, co-existent Barrett’s esophagus, and the need for esophageal dilation. Perforation secondary to dilation treatment can also contribute to the significant morbidity faced by patients with peptic strictures [7]. 

Barrett’s esophagus occurs in only 0.25% of children below 18 years presenting with upper gastrointestinal symptoms (dysphagia, epigastric pain, heartburn, vomiting, poor weight gain, etc.). Regarding etiology, there is a strong correlation between gastroesophageal reflux and Barrett’s esophagus. However, in children, symptoms may be relatively silent due to the impaired sensitivity of the columnar lining to acid. Gastroesophageal reflux disease is commonly seen in patients with motor and intellectual disabilities. Studies have estimated up to 10-25% of institutionalized patients have symptoms of vomiting, rumination, or regurgitation. Barrett’s esophagus in children with peptic strictures has not been well characterized, and its prevalence is not known [1].

Endoscopic balloon dilation is the first-line therapy for the management of esophageal stenosis as it is a safe and effective procedure [8]. Success rates reportedly range between 76% and 100% for esophageal strictures in children [9]. Following esophageal dilation, patients should be treated with proton pump inhibitors (PPIs) to promote healing and reduce the risk of stricture recurrence. PPIs promote esophagitis healing, and esophagitis healing, in turn, improves dysphagia and decreases dilation need in patients with peptic stricture [10]. Vigorously treating esophagitis with PPIs in patients with peptic strictures is a crucial part of the overall management [11]. Although the malignant transformation into adenocarcinoma in children is rare, proper surveillance of Barrett’s epithelium is vital in management [12].

## Conclusions

Although peptic stricture with Barrett’s esophagus is rare in children and typically presents with dysphagia, it needs to be included in the differential diagnosis of a child with the common symptom of vomiting in the setting of developmental delay. Barium esophagram and esophageal endoscopy with biopsy clarified the diagnosis in our patient, who had a vague history; he was then effectively treated with repeated balloon dilation and PPIs.
